# Macropinocytosis in *Gracilariopsis lemaneiformis* (Rhodophyta)

**DOI:** 10.3389/fpls.2023.1225675

**Published:** 2023-09-26

**Authors:** Haihong Chen, Yiyi Hu, Guanpin Yang, Pingping Li, Jingru Yin, Xiaoqing Feng, Qiong Wu, Jingyu Zhang, Baoheng Xiao, Zhenghong Sui

**Affiliations:** ^1^ Key Laboratory of Marine Genetics and Breeding, Ocean University of China, Qingdao, China; ^2^ Jiangsu Key Laboratory of Marine Bioresources and Environment, Jiangsu Ocean University, Lianyungang, China; ^3^ Co-Innovation Center of Jiangsu Marine Bio-industry Technology, Jiangsu Ocean University, Lianyungang, China; ^4^ Institutes of Evolution and Marine Biodiversity, Ocean University of China, Qingdao, China

**Keywords:** macropinocytosis, endocytosis, *Gracilariopsis lemaneiformis*, red alga, F-actin, PI3Ks, small GTPase

## Abstract

Macropinocytosis is an endocytic process that plays an important role in animal development and disease occurrence but until now has been rarely reported in organisms with cell walls. We investigated the properties of endocytosis in a red alga, *Gracilariopsis lemaneiformis*. The cells non-selectively internalized extracellular fluid into large-scale endocytic vesicles (1.94 ± 0.51 μm), and this process could be inhibited by 5-(N-ethyl-N-isopropyl) amiloride, an macropinocytosis inhibitor. Moreover, endocytosis was driven by F-actin, which promotes formation of ruffles and cups from the cell surface and facilitates formation of endocytotic vesicles. After vesicle formation, endocytic vesicles could be acidified and acquire digestive function. These results indicated macropinocytosis in *G. lemaneiformis*. Abundant phosphatidylinositol kinase and small GTPase encoding genes were found in the genome of this alga, while PI3K, Ras, and Rab5, the important participators of traditional macropinocytosis, seem to be lacked. Such findings provide a new insight into endocytosis in organisms with cell walls and facilitate further research into the core regulatory mechanisms and evolution of macropinocytosis.

## Introduction

Macropinocytosis is an endocytic process that allows cell to rapidly internalize large-scale extracellular fluid into the cell through macropinosomes, which bud off from the plasma membrane (PM) (Kerr and Teasdale, 2009; Lin et al., 2020). This large-scale pinocytosis plays an important role in a variety of functions, such as nutrient uptake, signal transmission, and immune response, and has a significant influence on individual development and disease occurrence in many species (Schneider et al., 2007; Commisso et al., 2013; Mishra et al., 2022).

In contrast with other endocytic processes, macropinocytosis can non-selectively internalize extracellular fluid and generate large vesicles generally >0.2 μm in size (Hacker et al., 1997). This process starts from F-actin-mediated extensions of the PM called ruffles that can form cups or flaps to contract and close to create macropinosomes (Condon et al., 2018; Kay et al., 2022). After formation, early stage macropinosomes can contact and fuse with other organelles in the endolysosomal system for maturation or can be recycled back to the PM (Norbury et al., 1995; Wang et al., 2014). Generally, macropinosomes can be identified through the internalization of fluid phase markers such as dextran and horseradish peroxidase due to the non-selective internalization of extracellular fluid in macropinocytosis (Lim and Gleeson, 2011).

Unique molecular properties related to the formation and maturation of macropinosomes are beginning to be elucidated. Phosphatidylinositol kinase plays an important role in these processes. For example, Class I PI3Ks catalyze the generation of phosphatidylinositol 3,4,5-phosphate [PtdIns(3,4,5)P3], which is involved in ruffle formation, contraction, and closure (Swanson and Araki, 2022). Furthermore, Class III PI3Ks (VPS34) catalyze the generation of phosphatidylinositol 3-phosphate (PtdIns3P), which regulates macropinosome maturation (Kerr and Teasdale, 2009). In cancer cells and amoeba, the regulatory molecular mechanism of small GTPase in macropinocytosis is relatively conserved. In the initial stage, Ras family GTPases regulate the downstream Rho family GTPase (Rho, Rac, and CDC42) aggregation inside the PM and interact with phosphoinositide 4,5-bisphosphate to activate WAVE/SCAR and WASP proteins. These proteins bind to PI(4,5)P2, G-actin, and the Arp2/3 complex to coordinate the assembly of the Arp2/3 complex and the transit of G-actin to F-actin. With the extension of the actin branch, the PM is extruded and further extended into ruffles to form macropinosomes (Kerr and Teasdale, 2009; King and Kay, 2019; Swanson and Araki, 2022). Following detachment from the PM, the new macropinosomes are rich in Rab5, and this GTPase activates VPS34 to synthesize PtdIns3P (Law et al., 2017). With increasing concentration of PtdIns3P, Rab5 is lost from macropinosome and Rab7 is recruited, indicating the transition from an early macropinosome to a mature macropinosome (Langemeyer et al., 2020).

Currently, macropinocytosis has been reported in protozoa and metazoa, but spontaneous occurrence has not been observed in plants and fungi (King and Kay, 2019). Regarding algae, although some unicellular algae such as the dinoflagellate *Alexandrium catenella* and bacillariophyta *Ulnaria ferefusiformis* exhibit some macropinocytosis features, like internalizing large molecular weight dextran (an established marker of macropinocytosis) via endocytosis, their specific physical and chemical properties and molecular mechanism have not been further investigated (Legrand and Carlsson, 1998; Annenkov et al., 2020). Therefore, the existence of macropinocytosis in algae has not yet been proven. In a recent study, endocytic activity was found to differ between the epidermal and non-epidermal cells of *Gracilariopsis lemaneiformis*, a model red alga, and the cells may use endocytosis to internalize extracellular carbohydrates to support growth (Chen et al., 2022). Therefore, this present study evaluated the endocytic properties of *G. lemaneiformis.* The results showed that endocytosis in *G. lemaneiformis* was similar to macropinocytosis.

## Results

### Endocytosis in *G. lemaneiformis* internalizes extracellular fluid and produces large-scale vesicles

**Figure 1:**
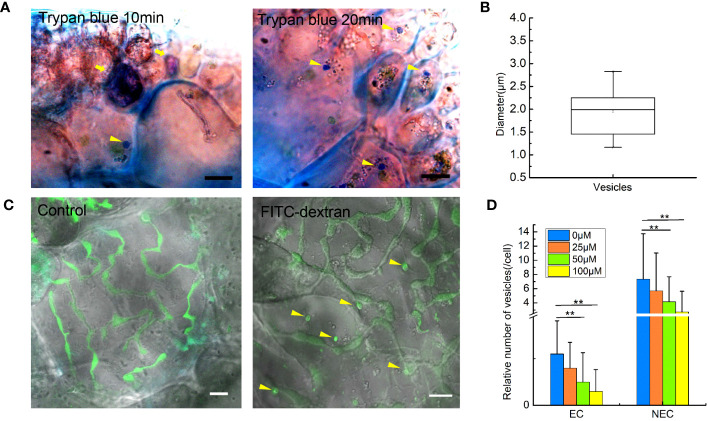
Extracellular fluid internalized by endocytosis in the cells of *Gracilariopsis lemaneiformis*. **(A)** Trypan blue internalized by cells. Dead cells are marked with tail arrows, while endocytic vesicles in living cells are indicated by arrow heads (internalized trypan blue). Bar=10 μm. **(B)** Statistical box diagram of the diameter of endocytic vesicles containing trypan blue. n=10. **(C)** Endocytosis of *G. lemaneiformis* internalizes FITC-dextran within 20 min; endocytic vesicles containing FITC-dextran (round or oval signal) in cells are marked with yellow arrows. Bar=10 μm. **(D)** Histogram of the number of endocytic vesicles in different cells affected by different concentrations of EIPA. EC, epidermal cell; NEC, non-epidermal cell. “**” indicate statistically significant differences by Student’s t-test (p < 0.01), n_EC_=30, n_NEC_=60.

To confirm if endocytosis in *G. lemaneiformis* can internalize extracellular fluid, the cell slices were treated with 0.4% trypan blue. As a dye used to identify cell activity, trypan blue could be effectively blocked by the PM of living cells but could enter the living cell through endocytosis. Therefore, trypan blue has been used to visualize pinocytosis in viable cells (Kerschbaum et al., 2021). In the present study, dead cells in the slices were stained dark blue, but the living cells were observed to internalize trypan blue into endocytic vesicles within 10 min (**Figure 1A**). After 20 min, number of vesicles coated with trypan blue further increased. Of note, the diameter of the dye-coated vesicles was larger than 0.2 μm; 1.94 ± 0.51 μm (**Figure 1B**). According to the size of endocytotic vesicles, we speculated that macropinocytosis exists in *G. lemaneiformis*. Therefore, *G. lemaneiformis* cells were treated with fluorescein-dextran, a well-established marker for detecting macropinocytosis (Hewlett et al., 1994; Commisso et al., 2013). When cells were incubated with 4 kDa FITC-dextran, FITC-dextran was internalized in the vesicles with diameters of approximately 2 μm, whereas the FITC signals (round or oval particles) were missing in the negative control (**Figure 1C**). Amiloride and its derivative EIPA can effectively inhibit macropinocytosis in animals and amoeba (West et al., 1989; Ivanov, 2008). When *G. lemaneiformis* was treated with different concentrations of EIPA, the number of endocytic vesicles decreased with increasing EIPA in a dose-dependent manner (**Figure 1D**).

### Cell ruffles and F-actin related to endocytosis in *G. lemaneiformis*

**Figure 2:**
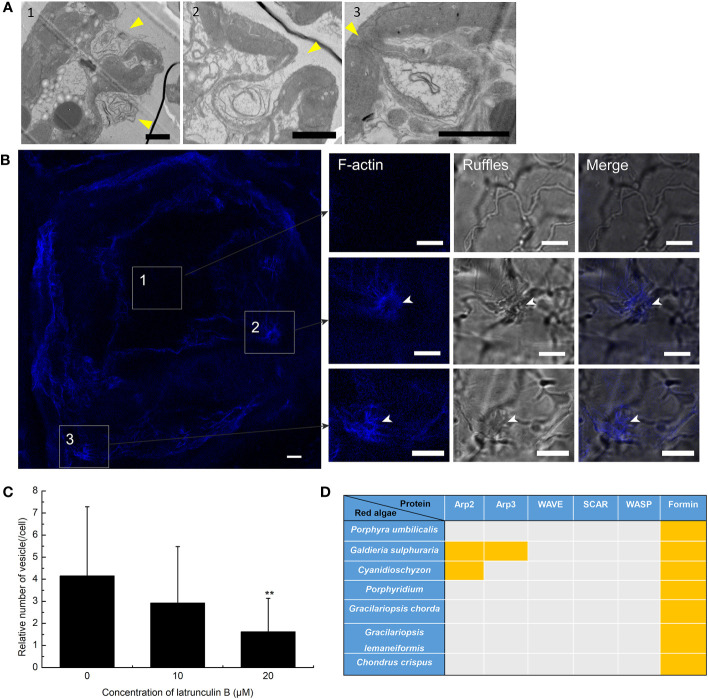
Plasma membrane (PM) ruffles and F-actin polymerization in the endocytosis of *Gracilariopsis lemaneiformis*. **(A)** Electron microscopy observation of *G. lemaneiformis* cell ruffles. Ruffles are marked by arrows. Numbers 1–3 show the different ruffle formation stages. Bar=2 μm. **(B)** F-Actin stained by Alexa Fluor Plus 405 phalloidin. F-Actin and ruffles were observed under DAPI filters and bright field, respectively. Numbers 1–3 show the different area of the cell. Bar=10 μm. **(C)** Statistical histogram of the number of endocytic vesicles in non-epidermal cells under different concentrations of latrunculin **(B)** “**” indicate statistically significant differences (p < 0.01) between the experimental group (25 μM or 50 μM) and control (0 μM) by Student’s t-test, n=40. **(D)** Proteins for F-actin polymeric regulation in the genomes of red algae. With the exception of *G. lemaneiformis*, relative proteins in other red algae refer to Goodson et al. (2021). Orange blocks indicate the presence of protein.

To further prove the presence of macropinocytosis in *G. lemaneiformis*, cell ruffles were observed, and the relationship between ruffles and F-actin was detected. To detect the PM cell ruffles, slices of *G. lemaneiformis* was observed using electron microscopy (**Figure 2**). Ruffles were formed by the cells and seemed to grow into cups, contacting and closing successfully into the cell body (**Figure 2A**). Cell ruffles were also observed on the cell surface by confocal microscopy (**Figure 2B**). F-Actin is one of the main regulating molecules for ruffle formation and closure. Therefore, cells were stained with phalloidin to observe F-actin. F-Actin was concentrated where ruffles occurred, and it aggregated at the edge of the cup ruffles, showing an “F-actin ring” (**Figure 2B**). When the cells were treated with latrunculin B, an inhibitor of F-actin polymerization, the endocytic activity of the cells significantly decreased with increasing dosage of this inhibitor (**Figure 2C**).

F-Actin polymerization cannot be achieved without the action of its key factors that regulate F-actin polymerization. The key proteins associated with F-actin aggregation were blasted in algal genomes. Arp2 and Arp3 are nucleating factors that can form the Arp2/3 complex to bind to microfilaments and promote the generation of new branched microfilaments (Goley and Welch, 2006). The Arp2/3 complex acts downstream of the WASP/SCAR/WAVE signal, forming nucleating branches on both sides of the existing parent filaments to generate a dense actin network (Seastone et al., 2001; Pollitt and Insall, 2009; Veltman et al., 2016; Davidson et al., 2018). However, Arp2, Arp3, and the WASP/SCAR/WAVE are generally lacking in red algal genomes (**Figure 2D**). Formin is another nucleating factor of F-actin that mediates the formation of unbranched actin filaments (Breitsprecher and Goode, 2013). Formins were found in *G. lemaneiformis* and other red algal genomes (**Figure 2D**), which suggested that F-actin polymerization was mediated by Formins.

### Acidification and digestion of *G. lemaneiformis* endocytic vesicles

**Figure 3:**
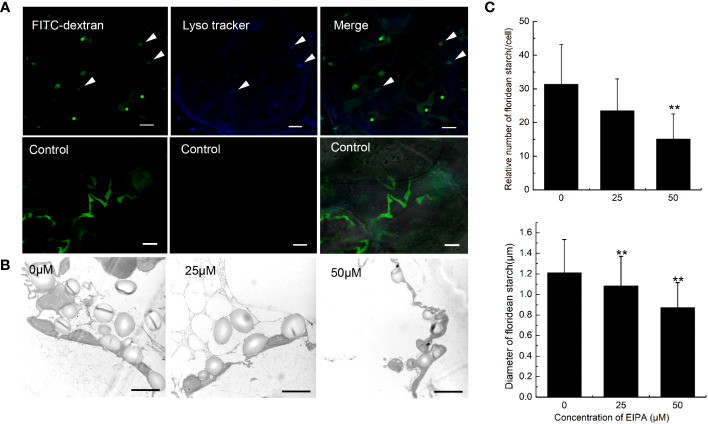
Acidification and digestive function of endocytic vesicles of *Gracilariopsis lemaneiformis*. **(A)** Acidification detection of endocytic vesicles in *G. lemaneiformis*. FITC-dextran and Lyso tracker were observed under FITC filter and DAPI filter, respectively. Bar=10 μm. **(B)** Electron microscopic image of floridean starch in *G. lemaneiformis* cells at different concentrations of 5-[N-ethyl-N-isopropyl] amiloride (EIPA). Bar=2 μm. **(C)** Statistical histograms of the number and diameter of floridean starch granules in the non-epidermal cells of *G. lemaneiformis* at different concentrations of EIPA. “**” indicate statistically significant differences (p < 0.01) between the experimental group (25 μM or 50 μM) and control group (0 μM) by Student’s t-test, n>3.

When macropinosome forms from ruffles, it is acidified via trafficking to the endosome/lysosome pathway (Racoosin and Swanson, 1993). To detect the acidification of endocytic vesicles, LysoTracker, a fluorescent dye that labels acidic organelles in living cells, was incubated with *G. lemaneiformis* cells. After 2 h, the vesicles marked by FITC-dextran were observed to be acidified (**Figure 3A**). Meanwhile, cells that internalized FITC-dextran were cultured and observed at different time points. The results showed that FITC-dextran signals gradually weakened and disappeared with time (**Supplementary Figure S1**). After 48 h, the FITC-dextran signals had almost disappeared. In contrast, the positive control (fixed cells) still had FITC-dextran signals even after 48 h (**Supplementary Figure S1**). FITC fluorescent signal is sensitive to pH changes, and fluorescence of FITC-dextran will be progressively quenched with decreasing pH (Geisow, 1984). This suggested that the endocytic vesicles of *G. lemaneiformis* were acidified. When macropinosome is acidified, it acquires the digestive function, which can degrade cargos to provide nutrients for cells (Kerr and Teasdale, 2009). Floridean starch is one of the main carbon sinks in *Gracilaia/Gracilariopsis* species (Viola et al., 2001; Chen et al., 2022). In the current study, the floridean starch content of cells was examined after inhibition of endocytosis in *G. lemaneiformis*. The results indicated that the floridean starch content of the cells experienced a significant reduction subsequent to a 1-week incubation period with EIPA (**Figures 3B, C**). This suggested that inhibition of endocytosis in *G. lemaneiformis* led to a decrease in the presence of energy storage substances within the cells.

### Proteins of phosphatidylinositol kinase and small GTPase in the *G. lemaneiformis* genome

**Figure 4:**
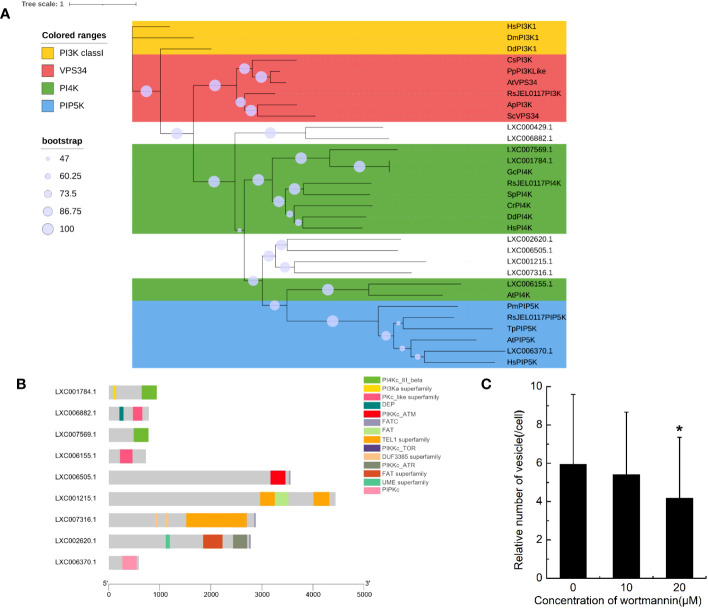
The genes encoding phosphatidylinositol kinase in *Gracilariopsis lemaneiformis* genome. **(A)** Phylogenetic analysis of phosphatidylinositol kinase members. The tree was constructed using a maximum likelihood method, and the bootstrap values (1,000 replicates) are shown on each node. Hs, *Homo sapiens*; Dm, *Drosophila melanogaster*; Dd, *Dictyostelium discoideum*; Sc, *Saccharomyces cerevisiae*; Cs, *Coccomyxa subellipsoidea*; Pp, *Physcomitrium patens*; At, *Arabidopsis thaliana*; Gc, *Gracilariopsis chorda*; Cr, *Capsella rubella*; Sp, *Schizosaccharomyces pombe*; Tp, *Thalassiosira pseudonana*; Pm, *Plasmodium malariae*; Ap, *Aureobasidium pullulans*; RsJEL0117, *Rhizoclosmatium* sp. JEL0117. **(B)** Conserved domain of the proteins that blasted by phosphatidylinositol kinase from *G. lemaneiformis* protein database. **(C)** Statistical histogram of the number of endocytotic vesicles in non-epidermal cells under different concentrations of wortmannin. “*” indicate statistically significant differences (p < 0.05) between the experimental group (10 μM or 20 μM) and control group (0 μM) by Student’s t-test, n=34.

The domains of PI3Ks were searched in genome of *G. lemaneiformis* using HMMER (**Supplementary Table S1**). Protein containing the Ras binding domain (RBD) appeared to be lacking (**Supplementary Table S1**). Additionally, proteins containing the p85B domain and C2 domain were also lacking. Meanwhile, a set of protein sequences of phosphatidylinositol kinase, which contained class I PI3K, VPS34, PI4K, and PIP5K, were used to blast the protein database of *G. lemaneiformis*. A total of 10 proteins were found in *G. lemaneiformis* and the sequence sets (**Supplementary File 1**) containing these proteins were used to construct a phylogenetic tree. The results showed that none of the proteins of *G. lemaneiformis* were clustered into the branches of class I PI3K and VPS34 (**Figure 4A**). LXC001784.1 and LXC007569.1 were clustered with PI4Ks, and these proteins both contained the domain of PI3_PI4_kinase (**Supplementary Table S1**; **Figure 4A**). Although LXC001784.1 contained a PI3Ka domain related to substrate presentation in PI3Ks (**Supplementary Table S1**; **Figure 4B**), conserved domain analyses showed that LXC001784.1 and LXC007569.1 had a catalytic domain of PI4K (**Figure 4B**). In addition, LXC006155.1 was clustered with the PI4K of plants and had a Pkc_like superfamily catalytic domain, a domain that has the catalytic activity of phosphokinase (**Figures 4A, B**). In addition, LXC006370.1 was clustered with PIP5K and had a PIPKc domain, which is a conserved core region in the PIP5K family (**Figures 4A, B**). Wortmannin, an inhibitor of phosphatidylinositol kinases, was incubated with *G. lemaneiformis* to investigate whether phosphatidylinositol kinases participate in *G. lemaneiformis* endocytosis. After treatment with 20 μM wortmannin, the endocytic activity of cells was significantly decreased, while 10 μM had no significant effect (**Figure 4C**).

**Figure 5:**
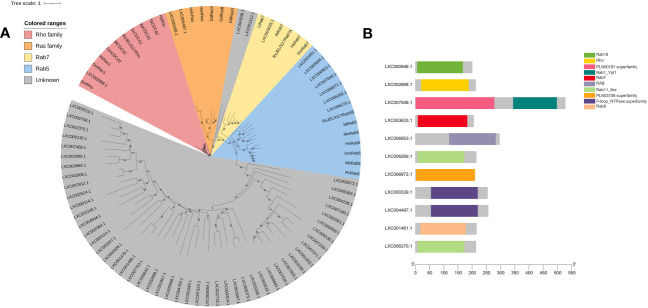
Phylogenetic analysis of small GTPase of *Gracilariopsis lemaneiformis*. **(A)** The tree was constructed using a maximum likelihood method, and the bootstrap values (1,000 replicates) are shown on each node. At, *Arabidopsis thaliana*; Hs, *Homo sapiens*; Dd, *Dictyostelium discoideum*; Dm, *Drosophila melanogaster*; Li, *Leishmania infantum*; Nt, *Nicotiana tabacum*; Pc, *Procambarus clarkia*; Ms, *Manduca sexta*; Bt, *Bos taurus*; Sc, *Saccharomyces cerevisiae*; Ta, *Trichoderma atroviride*; Mn, *Monoraphidium neglectum*; Ap, *Aureobasidium pullulans*; RsJEL0117, *Rhizoclosmatium* sp. JEL0117. **(B)** Conserved domain of the proteins that blasted by small GTPase from *G. lemaneiformis* protein database.

In this study, the hidden Markov model of the Ras family (PF00071) and a sequence set of Ras superfamily members were used to blast the genome of *G. lemaneiformis* and to construct a phylogenetic tree using the sequence set containing the protein sequences being blasted (**Supplemental File 2**). The results showed that LXC002888.1 was clustered into one branch with Rho, Rac1, and CDC42, which has a Rho domain (**Figures 5A, B**). Meanwhile, Rab7, a marker of a mature macropinosomes, was also clustered with the protein of *G. lemaneiformis*, and the protein has the Rab7 domain. LXC000339.1 and LXC004497.1 were clustered into one branch with the Ras family (bootstrap=99), and both of them has a conserved domain of P-loop NTPase superfamily. Although Rab5 that was enriched in the early macropinosomes was clustered together with seven proteins of *G. lemaneiformis* (bootstrap=94), the proteins do not contain the typical domain of Rab5 but contain the domain of other members in Rab family such as Rab11-like or Rab18.

## Discussion

Many features of *G. lemaneiformis* suggest that macropinocytosis occurs in this alga. The large size of the endocytic vesicles is one of the direct forms of evidence for macropinocytosis. Clathrin- and caveolae-mediated endocytosis produce vesicles, usually smaller than 0.2 μm (Cohen et al., 2004; Kaksonen and Roux, 2018). However, endocytosis in *G. lemaneiformis* can produce vesicles with a diameter larger than 0.2 μm. Moreover, the endocytic vesicles of *G. lemaneiformis* can form from the ruffles of the PM and are regulated by F-actin polymerization, which is consistent with the characteristics of macropinosome formation in cancer cells and *Dictyostelium* sp. (Kay et al., 2022; Lambies and Commisso, 2022). Phagocytosis can also produce large-scale endocytic vesicles that are often >0.2 μm, and the phagosome usually produces from PM ruffles, regulated by F-actin polymerization (Lee and Knecht, 2002). Phagocytosis requires ligands on solid particles to bind to membrane receptors in order to initiate phagocytosis, whereas macropinocytosis involves non-selective extracellular fluid uptake (Kerr and Teasdale, 2009). In *G. lemaneiformis*, endocytosis can non-selectively internalize extracellular fluid without the need for solid mediation, which is more similar to the characteristics of macropinocytosis. In addition, the endocytic vesicles in *G. lemaneiformis* were acidified, suggesting that the fate of endocytic vesicles of *G. lemaneiformis* was similar to that of traditional macropinosomes. In cancer cells, and amoeba, cells usually obtain extracellular nutrients by macropinocytosis to sustain growth (Commisso et al., 2013; King and Kay, 2019; Zhang et al., 2022). NEC is the sink cell in the branches of *G. lemaneiformis* (Chen et al., 2022), which had more endocytic vesicles than EC (**Figure 1D**). In the current study, the inhibition of endocytosis activity reduced the content of floridean starch in the cells of *G. lemaneiformis* (**Figure 3B**), further suggesting that endocytosis is related to energy storage function of cells. These results suggest that the function of endocytosis in *G. lemaneiformis* is similar to that of macropinocytosis.

It must be noted that although both traditional macropinocytosis and endocytosis in *G. lemaneiformis* are regulated by F-actin, mechanisms of F-actin polymerization seem to be different. In animals, Arp2/3 is the main nucleating factor for F-actin polymerization, while formins have a weak nucleation activity and may mediate filament elongation synergies with the Arp2/3 complex in actin assembly (Junemann et al., 2016). However, the absence of Arp2, Arp3, and their upstream-related proteins in the genome of *G. lemaneiformis* suggests that F-actin polymerization in this alga may not depend on the Arp2/3 nucleation system. The evolutionary relationship between algae and plants is closer than that of animals. In higher plants, actin can be assembled by formins without the Arp2/3 complex. For example, in the central cells of *Arabidopsis thaliana*, F-actin polymerization is Arp2/3 independent but controlled by formins (Ali and Kawashima, 2021). Algae are generally abundant in formins (**Figure 2**), suggesting that formins may be involved in endocytosis pathway of *G. lemaneiformis* by regulating F-actin polymerization.

Although class I PI3Ks play an important role in traditional macropinocytosis, the encoding genes of class I PI3Ks appear to be absent in *G. lemaneiformis*. Notably, the catalytic subunit of class I PI3Ks contain an RBD in which Ras can activate class I PI3Ks through combining this domain to regulate the macropinocytic process in *Dictyostelium* sp. and mammalian cells (Bar-Sagi and Feramisco, 1986; Yang et al., 2012; King and Kay, 2019), while this domain is absent in *G. lemaneiformis*. In addition, some proteins in *G. lemaneiformis* clustered with Ras and Rab5, while these proteins lack the typical domains of Ras and Rab5 (**Figure 5B**). Although some of crucial genes could be part of the pan-genome, it is more likely that genes of class I PI3Ks and Rab5 are missing in *G. lemaneiformis*. According to previous study, class I PI3Ks are generally absent in plants, fungi, and algae (Vanhaesebroeck et al., 2012; Kriplani et al., 2015). Rab5 has also been found to be absent in modern red algae (Petrželková and Eliáš, 2014). These all suggested that the mechanisms of macropinocytosis in *G. lemaneiformis* quite different from those in animal cells and macropinocytosis might have been rewired in rhodophytes.

PI4K can also regulate the polymerization of F-actin. The F-actin of sperm increases when PI4K is activated by PI4K activator and spermatin (Etkovitz et al., 2007). Additionally, PI4Ks were found to have a regulatory effect on plant endocytosis (Sorensen et al., 1998). Furthermore, wortmannin can bind the 110-kDa catalytic subunit of PI3Ks, with specific inhibition of PI3K activity at nanomole levels. However, micromolar dosage of wortmannin can also inhibit the activity of PI4Ks (Etkovitz et al., 2007). In *G. lemaneiformis*, 20 μM of wortmannin significantly decreased the activity of endocytosis, while a dose of 10 μm had no significant effect. In addition, PIP5K was found in the *G. lemaneiformis* genome. PI(4,5)P2 is produced from PtdIns 4P by PIP5K, and PtdIns 4P is synthesized from phosphatidylinositol (PtdIns) by PI4K. In macrophages, PI(4,5)P2 levels spike in the macropinocytic cups of macrophages, revealed by subcellular localization (Welliver and Swanson, 2012). These results implied that PI4Ks were related to endocytosis in this alga.

Macropinocytosis was first discovered approximately 100 years ago; it has mainly been described in animals (Lewis, 1931) but rarely reported in plants, fungi, and algae. The presence of cell walls has been considered to impede the occurrence of macropinocytosis in these species (King and Kay, 2019). Algae share common evolutionary features with plants, including a carbohydrate-rich cell wall (Popper et al., 2011). Macropinocytosis in *G. lemaneiformis* suggests that the cell wall is not an essential requirement for preventing macropinocytosis in species with cell walls. Current fossil research findings have revealed that Rhodophyta might have originated 1.6 billion years ago (Bengtson et al., 2017), indicating that they are one of the oldest organisms on Earth. The detection of macropinocytosis in *G. lemaneiformis* signifies the origin of this endocytic pathway in the common ancestors of Rhodophyta, protists, and metazoans. In view of a common evolutionary origin, the discovery of macropinocytosis in red algae provides an opportunity to compare the macropinocytosis of Rhodophyta with that of animals, which can have immense significance for revealing the core and conservative components, and mechanisms of macropinocytosis.

## Methods

### Plant materials and growth conditions

*Gracilariopsis lemaneiformis* were collected from aquaculture areas of Rongcheng, Shandong, China, and cultured in Provasoli’s medium (Pflugmacher et al., 1999) at 20°C under a 12-h/12-h light/dark cycle and illumination intensity of 30 μmol photons m^−2^ s^−1^.

### Slice preparation and treatment

The shoot apices of the algae branches (~3 cm in length) were harvested and sliced using razor blades. To detect the non-selective internalized extracellular fluid by endocytosis, approximately 50 slices were incubated with 500 μL of 0.04% trypan blue (Solarbio, Cat. C0040) for 10 and 20 min, and the vesicles containing trypan blue in living cells were determined. Approximately 50 slices were also incubated with 500 μL of 0.1 mg/mL FITC-dextran (Xi’an ruixi Biological Technology Co., Ltd., Cat. R-FD-001) to determine whether they internalized dextran. For determining the inhibition of endocytosis, different inhibitors [5-(N-ethyl-N-isopropyl)] amiloride (EIPA) (MedChemExpress, Cat. HY-101840), lacunculin B (Aladdin, Cat. L275457), and wortmannin (MedChemExpress, Cat. HY-10197) were incubated with slices for 1 h, and then, the slices were incubated with the mixture of inhibitors and FITC-dextran for 50 min. After fixing in 4% paraformaldehyde (Solarbio, Cat. P1110) at 4°C for 2 h, the slices were observed using a laser confocal microscope (Nikon A1R HD25, Japan). For F-actin staining, the slices were fixed overnight in 1 mL of 4% paraformaldehyde at 4°C and washed three times using 0.1% Triton X 100 (1× PBS). The slices were then stained with 100 μL of 0.67 µM Alexa Fluor Plus 405 Phalloidin (Invitrogen, Cat. A30104) for 5 h. For detecting vesicle acidification, the slices were incubated with 500 μL of 0.1 mg/mL FITC-dextran for 50 min and then with 200 μL of 1 μM Lysotracker Blue DND 22 (Invitrogen, Cat. L7525) for 1 h. After washing, the slices were observed using laser confocal microscopy.

### Gene research and identification

Protein sequences were download from the National Center for Biotechnology Information (https://www.ncbi.nlm.nih.gov/), and the sequence sets were searched in the *G. lemaneiformis* genome using Blastp. Hidden Markov models of domain sequences were downloaded from Pfam (http://pfam-legacy.xfam.org/), and HMMER was used to search the gene family members from the alga genome. The conserved domain was predicted by the CD search of the National Center for Biotechnology Information (https://www.ncbi.nlm.nih.gov/) and Smart (http://smart.embl-heidelberg.de/). Multiple sequence alignment was performed with Mafft (Katoh and Standley, 2013), and a phylogenetic tree was constructed using Iqtree (Minh et al., 2020).

### Electron microscopy and quantification of starch granules

Shoot apices (3 mm) were harvested for ultrastructural observation. The electron microscopy samples were prepared and observed as described by Chen et al. (2022). In brief, the samples were fixed in 1% osmium tetroxide after being fixed overnight in 2.5% glutaraldehyde. Then, samples were dehydrated with ethanol treatments and embedded in Epon 812. After being cured, the samples were cut with a Reichert-Jung Ultracut E Ultramicrotome (Germany) and were observed under a JEOL JEM-1230 transmission electron microscope (Japan). The number of starch granules and particle diameters of cells were measured using Image J.

### Statistical analysis

Statistical analysis of the obtained data was performed using SPSS. Statistical differences were evaluated by Student’s *t*-test for two-group comparison.

## Data availability statement

The original contributions presented in the study are included in the article/**Supplementary Material**, further inquiries can be directed to the corresponding author/s.

## Author contributions

HC and ZS designed the research. HC, YH, PL, JY, XF QW, JZ, and BX performed the research. YH developed the statistical methods. HC and ZS analyzed the data. HC, ZS, and GY wrote the article with approval from all authors. All authors contributed to the article and approved the submitted version.

## Supplementary material

Supplemental File 1 Sequence set of phosphatidylinositol kinase.

Supplemental File 2 Sequence set of small GTPase.
