# Supplementary material for: Macropinocytosis in Gracilariopsis lemaneiformis (Rhodophyta)
Source: Front Plant Sci. 2023 Sep 26;14:1225675. doi: 10.3389/fpls.2023.1225675 (PMC10562585; doi:10.3389/fpls.2023.1225675)
Supplement: Supplemental File 1 — Sequence set of phosphatidylinositol kinase. [file DataSheet_1.pdf]

Supplemental figure and table

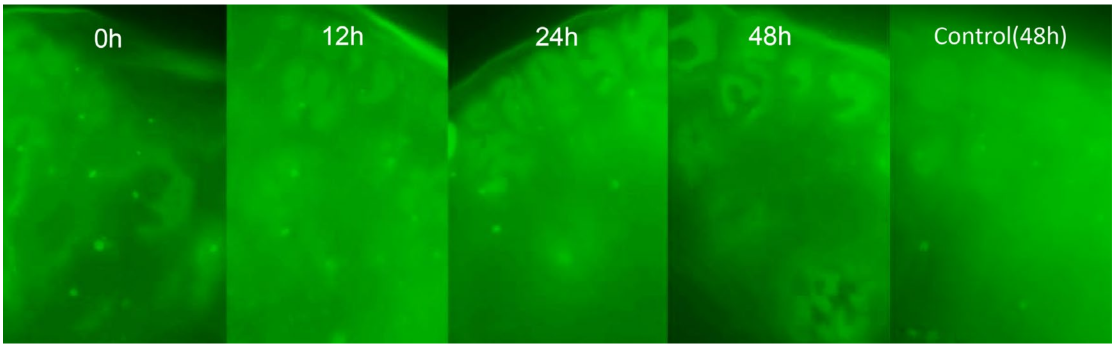

Figure S1 FITC-dextran in vesicles at different time. The cells were incubated with 0.05 mg/mL FITC-dextran for 1 h at 20°C. Then, the cells were cultured with seawater at 20°C in dark and observed after 0, 12, 24, and 48 h. The control cells were incubated with FITC-dextran for 1 h at 20°C, treated with fixer (methanol:acetone=1:1 fixed for 60 s) and then with seawater at 20°C in dark for 48 h.

Table S1 Alignment of the homeodomain of PI3K gene in the genome of *Gracilariopsis lemaneiformis*

Domain homologs	Gene	Accession ID
PI3K_RBD	-	PF00794
PI3K_p85B	-	PF02192
PI3K_C2	-	PF00792
PI3Ka	LXC001784.1	PF00613
PI3_PI4_kinase	LXC007316.1, LXC006505.1, LXC002620.1, LXC001784.1, LXC007569.1, LXC006155.1, LXC006882.1, LXC001215.1	PF00454
